# The value of structured data elements from electronic health records for identifying subjects for primary care clinical trials

**DOI:** 10.1186/s12911-016-0239-x

**Published:** 2016-01-11

**Authors:** Mohammad B. Ateya, Brendan C. Delaney, Stuart M. Speedie

**Affiliations:** 1 University of Michigan Health System, University of Michigan MCIT, 24 Frank Lloyd Wright Dr., Lobby J Suite 4000, Ann Arbor, MI 48105 USA; 2 Imperial College London, London, UK; 3 Institute for Health Informatics, University of Minnesota, Minneapolis, MN USA

**Keywords:** Primary care research, Eligibility criteria, Electronic health records, Structured data

## Abstract

**Background:**

An increasing number of clinical trials are conducted in primary care settings. Making better use of existing data in the electronic health records to identify eligible subjects can improve efficiency of such studies. Our study aims to quantify the proportion of eligibility criteria that can be addressed with data in electronic health records and to compare the content of eligibility criteria in primary care with previous work.

**Methods:**

Eligibility criteria were extracted from primary care studies downloaded from the UK Clinical Research Network Study Portfolio. Criteria were broken into elemental statements. Two expert independent raters classified each statement based on whether or not structured data items in the electronic health record can be used to determine if the statement was true for a specific patient. Disagreements in classification were discussed until 100 % agreement was reached. Statements were also classified based on content and the percentages of each category were compared to two similar studies reported in the literature.

**Results:**

Eligibility criteria were retrieved from 228 studies and decomposed into 2619 criteria elemental statements. 74 % of the criteria elemental statements were considered likely associated with structured data in an electronic health record. 79 % of the studies had at least 60 % of their criteria statements addressable with structured data likely to be present in an electronic health record. Based on clinical content, most frequent categories were: “disease, symptom, and sign”, “therapy or surgery”, and “medication” (36 %, 13 %, and 10 % of total criteria statements respectively). We also identified new criteria categories related to provider and caregiver attributes (2.6 % and 1 % of total criteria statements respectively).

**Conclusions:**

Electronic health records readily contain much of the data needed to assess patients’ eligibility for clinical trials enrollment. Eligibility criteria content categories identified by our study can be incorporated as data elements in electronic health records to facilitate their integration with clinical trial management systems.

## Background

Research studies are central to advancing the science of health care. Randomized controlled trials (RCTs) are the most reliable means of estimating the differences between healthcare interventions [1]. Randomization, allocation concealment and blinding of outcome measurement are the fundamental tasks in an RCT [1]. Observational and other types of quasi-experimental designs also have a major role to play in the research endeavor when meeting the requirements for an RCT is not possible, or in determining if predicted results are obtained in routine clinical practice [2].

Clinical trials have typically been conducted in large academic medical centers although most patient care is done in community settings [3, 4]. In 2008, approximately 62 % of the 1.1 billion ambulatory care visits in the United States were performed in primary care practices [5]. If a healthcare system is to be genuinely evidence-based, much greater emphasis needs to be placed on clinical research in the primary care setting, and much sooner in the translational pathway. Primary care represents an important entry point for new findings into the community, and patients seen in primary care practices can benefit from access to experimental treatments faster if research is conducted in primary care settings [6].

Over the past 20 years, an increasing number of ‘pragmatic’ or late translational RCTs have been conducted by practice-based research networks in Europe and the North America. However, such studies are challenging to conduct and resource-intensive [6]. In particular primary care physicians see patients with a wide spectrum of medical conditions and even the most common medical conditions constitute a small percentage of all primary care contacts. Thus the number of patients who could be recruited from a single clinic is relatively small compared to a hospital or specialty setting [7]. There is a need to improve the effectiveness and efficiency of trials in primary care by making better use of the available patient clinical information such as that in their electronic health records (EHRs) [7].

Eligibility criteria specify the population for a study. They drive clinical trial recruitment, selecting subjects for observational studies, and generalizability of results [1]. For RCTs recruitment of enough subjects for a trial in an acceptable time frame is a difficult but important task. A recent review found that less than 31 % of RCTs were able to meet their original recruitment target on time [8].

Eligibility criteria are usually expressed as descriptive text rather than combinations of discrete clinical data elements. This makes them less readily amenable to a computable representation or a set of rules and associated data elements that can be implemented as a computer algorithm. Formally computable (structured) representation of eligibility criteria is increasingly useful in the era of EHRs, to facilitate various research functions including evaluating feasibility, cohort identification and trial recruitment [9].

There are several efforts underway to share clinical trials protocols including eligibility criteria. These include the National Library of Medicine’s ClinicalTrials.gov, the European Clinical Trials Database (EudraCT), and the UK Clinical Research Network Study Portfolio (http://public.ukcrn.org.uk/search/). However none of these require standard or computable representations of eligibility criteria. One effort to create an ontology of clinical research is the Human Studyome Project’s Ontology of Clinical Research (OCRe), which aims to capture the design, process, and results of clinical research into a standardized-format to support wide-scale data queries, aggregation, and reuse of clinical research studies [10]. Another in the area of primary care based research is the European FP7 Translational Research and Patient Safety in Europe project (TRANSFoRM - www.transformproject.eu) that has developed the clinical research information model (CRIM) which may also be used for representing eligibility criteria that can be used in different EHRs in primary care practices to identify patients eligible for research studies [11].

Computable representations of eligibility criteria are an important cornerstone in the broader work towards creating a standards-based, computable, study protocol model. This effort is beyond the electronic sharing of text-based protocol documents. A computable study protocol would have many benefits at various stages of clinical research but current efforts lack standardization [12]. The Clinical Data Interchange Standards Consortium (CDISC) is in the process of developing a set of standards for the expression of clinical trial protocols, including eligibility criteria in a computable format, however this is a complex task as it requires standardization or at a minimum an approach to standardization of data elements across the whole biomedical domain [13].

EHRs contain a wealth of patient data that can potentially be used as a source for wider-scale screening of patients for study enrollment. In 2012, 44 % of non-federal acute care hospitals in the US had adopted at least a basic EHR, and 85 % of those possessed a certified EHR [14].

The potential for using EHR data for study screening has been demonstrated with EHR driven clinical trial alerts (CTA) resulting in a 10-fold increase in study referrals [15].

A major issue in using computable criteria to identify study subjects using EHR data is that there is a semantic “gulf” between clinical data in the EHR and current expressions of clinical trial eligibility criteria [16].

Recent studies have addressed this issue in terms of content and eligibility determination [17]. Van Spall et al. examined exclusion criteria for RCTs published in major medical journals and classified them into criteria based on consent, age, sex, medical comorbidities, medication-related, socioeconomic status, communication or language barriers, ethnicity, and participation in other trials [18]. Ross et al. analyzed a random sample of 1000 eligibility criteria and reported that 71 % of criteria specified patient clinical attributes, 34 % of criteria specified treatments or interventions participants have received or will receive, and 4 % of criteria specified patient behavior [19]. Using semantic types from the Unified Medical Language System (UMLS), Luo et al. identified 27 semantic classes in a sample of 2718 eligibility criteria sentences [20]. Weng et al. categorized the same eligibility criteria sample from the study of Ross et al. based on content using 23 of the 27 classes identified by Luo et al. [21]. Köpcke et al. analyzed eligibility criteria from 15 clinical trials performed in 5 tertiary care hospitals in Germany. Köpcke et al. categorized the eligibility criteria based on the semantic categories identified by Luo et al. and found distribution among semantic categories similar to the results of Luo et al. [22, 23]. A similar more recent study by Doods et al. evaluated eligibility criteria from 40 clinical trials to identify the most common data elements used for patient identification in pharmaceutical clinical trials. Doods et al. compared their categorization of eligibility criteria to the categorization of Luo et al. and Köpcke et al. and reported similar results to the comparison studies [24].

This study investigates the potential use of clinical data in the EHR to facilitate automated screening of patients who might be candidates for primary care clinical studies. First it attempts to quantify the proportion of eligibility criteria that can be addressed with structured data or information typically found in an EHR in order to explore the feasibility of automated screening of patients for study eligibility. The second goal is to categorize eligibility criteria and their criteria elemental statements from trials in the primary care domain based on content, comparing the results with the work of Weng et al., and Köpcke et al. to validate our findings and characterize the breadth, depth, and variety of clinical data present in primary care clinical research eligibility criteria.

## Methods

A set of primary care study descriptions including eligibility criteria were extracted from the publicly available UK Clinical Research Network (UKCRN) Study Portfolio website in 2011. The UKCRN Study Portfolio is a database of high-quality studies eligible for consideration for support from the UK National Institute for Health Research (NIHR) Clinical Research Network. The UKCRN Study Portfolio defines primary care as the “care that describes range of services that are normally the first point of contact for participants” [25]. All studies classified as primary care related were identified and eligibility criteria descriptions including both inclusion and exclusion criteria were extracted from those studies. Studies added to the UKCRN Study Portfolio website after 2011 were not added to our data set since the number of studies was deemed sufficient for the research objectives.

Since eligibility criteria may contain multiple components, the criteria that contained more than one component were broken down into criteria elemental statements (CES) by one of the authors (MA), where each elemental statement is a single, simple statement that is used to determine eligibility. For example, “individuals who received a clinical diagnosis of bipolar disorder or who have experienced a first episode of mania within the last 5 years” was broken into two CES: “individuals who received a clinical diagnosis of bipolar disorder” and “who have experienced a first episode of mania within the last 5 years”. Logical connectors such as “and” and “or” were considered to mark boundaries of a CES for the purpose of this study. A negation modifier “NOT” was added to identify exclusion criteria.

Two independent expert raters (MA and SS) classified each CES based on whether or not structured data items in an EHR could be used to determine if the CES was true or false for a specific patient. If, in the opinion of a rater, such structured items were typically present in EHRs of which they had knowledge, they were labeled as “likely present” otherwise “unlikely”. Inter-rater disagreements were discussed until 100 % agreement was reached. Examples of CES that are readily available in the EHR are those such as “age >18”, “female”, “currently on lisinopril”, and “previously undergone total knee replacement.” Examples of CES unlikely to be present in an EHR are criteria related to patient preference such as “Women who at study entry, plan to have their child adopted”, or patient’s ability to give consent such as “Inability to give informed consent”. We quantified the proportion of CES that are likely present in a typical integrated EHR in total and per each study.

CES were also classified using categories similar to the categorizations used by Weng et al. (Table 1). When CES could not be classified under one of the categories identified by Weng et al., a label was manually applied to it by raters, and then labels were consolidated into 4 new categories based on agreement between raters.

**Table 1:** Eligibility criteria classification categories based on content

Category	Example
Addictive behavior^a^	NOT (Suspected abuse of alcohol or other drug)
Address^a^	Residents of selected deprived neighborhoods in the city of Sheffield
Age^a^	Aged greater than 40 years
Allergy^a^	NOT (Subjects with known hypersensitivity to sulfonamides)
Cancer^a^	NOT (Patients with lung cancer)
Capacity^a^	Dependence in functional activities of daily living requiring assistance
Compliance with protocol^a^	Able to comply with the intervention and all study procedures
Consent^a^	Consent to participate in the trial
Device^a^	NOT (metal implants)
Diagnostic or lab results^a^	Patients must have a measured post-albuterol/salbutamol FEV1/FVC ratio of <0.70 at Screening
Disease stage^a^	NOT (with stage 0 or IV prolapse)
Disease, symptom and sign^a^	Newly diagnosed depression
Enrollment in other studies^a^	NOT (Patients currently in, or have been in another clinical trial in the previous 3 months)
Gender^a^	Males
Life expectancy^a^	Life expectancy must be at least 5 years
Literacy or spoken language^a^	Be able to communicate in English sufficiently to complete questionnaires
Medication^a^	On maximum dose of oral anti-diabetic drugs
Organ or tissue status^a^	NOT (Organic brain damage)
Patient preference^a^	Wish to participate
Pregnancy conditions^a^	Pregnancy 8–18 weeks
Receptor status^a^	Hormone receptor status not specified
Special patient characteristics^a^	Owner of mobile phone
Therapy or procedure^a^	All patients undergoing an elective primary total knee arthroplasty for osteoarthritis
Practice Geographic location	NHS general practices within a 50 mile radius of Manchester and Nottingham in England
Practice/practitioner specialty	NOT (under the care of the specialist mental health team)
Practitioner preference	Locality specialist who will support the participation of the practice and the implementation of standard guidelines across the participating practices.
Caregiver attribute	CARERS: Fluent in English

^a^Categories were also used by Weng et al.

## Results

251 primary care studies were identified from the UK Clinical Research Network Study Portfolio. Eligibility criteria were retrieved for 228 studies, and were not available for the other 23 studies. Eligibility criteria were decomposed into 2619 eligibility criteria elemental statements (CES). 25 CES were excluded because they were malformed. The number of CES per study ranged from 1 to 68 CES (mean = 11, median = 10). A number of the CES were similar from study to study especially those involving gender or age.

74 % of the CES were considered likely associated with structured data in an EHR. For 14 % of the studies, all of their associated CES in their eligibility criteria could be addressed with structured data likely to be present in an EHR. 33 % of studies had less than 100 % but 80 % or more of their CES addressable with data present in the EHR. 32 % of studies had less than 80 % but 60 % or more of their CES addressable with data present in the EHR. 14 % of studies had less than 60 % but 40 % or more of their CES addressable with data present in the EHR. 4 % of studies had less than 40 % but 20 % or more of their CES addressable with data present in the EHR. 3 % of studies had less than 20 % of CES addressable (Table 2).

**Table 2:** Percentages of likely present CES per study

Percentage of Likely Present CES per Study	Number of Studies (%)
=100 %	31 (14)
≥80–<100 %	76 (33)
≥60–<80 %	74 (32)
≥40–<60 %	31 (14)
≥20–<40 %	10 (4)
≥0–<20 %	6 (3)
Total	228 (100)

CES were further classified into more granular content categories (Table 3) based on the categories used by Weng et al. 36 % of CES were classified as “disease, symptom or sign”, 13 % were classified as “therapy or procedure”, 10 % were classified as “medications”, and 7 % classified as “age”. These categories are not mutually exclusive and also add up to more than 100 %. For example eligibility criteria elemental statements classified as “medication” and “device” also fall under the category “therapy or surgery”, and 30 out of the 42 eligibility criteria elemental statements classified as “allergy” also fall under the category “medications”.

**Table 3:** Eligibility criteria classification based on semantic categories

CES Category	This Study^a^ % (n)	Weng et al. %	Köpcke et al. %
Medical Condition (Health Status)			
Disease, symptom and sign	36 % (944)	28 %	22.52 %
Pregnancy conditions	4 % (110)	3 %	5.24 %
Allergy	2 % (42)	1 %	5.95 %
Disease stage	1 % (20)	6 %	2.27 %
Cancer	0.3 % (9)	12 %	3.4 %
Organ or tissue status	0.1 % (2)	1 %	5.38 %
Life expectancy	0.1 % (2)	0 %	0.38 %
Treatment or Healthcare			
Therapy or surgery	13 % (352)	15 %	10.2 %
Medication (Pharmaceutical substance or drug)	10 % (255)	17 %	7.37 %
Device	0.5 % (14)	0 %	0 %
Diagnostic or Lab Tests			
Diagnostic or lab results	5 % (129)	14 %	19.41 %
Receptor status	0 % (0)	0 %	0 %
Demographics			
Age	7 % (196)	2 %	2.69 %
Special patient characteristics	5 % (139)	1 %	0.42 %
Literacy or spoken language	3 % (83)	0 %	0.28 %
Gender	2 % (53)	0 %	1.27 %
Address	1 % (25)	1 %	0 %
Ethical Considerations			
Preference	5 % (133)	1 %	0.57 %
Consent	4 % (94)	1 %	2.55 %
Capacity	3 % (69)	1 %	3.54 %
Gender	2 % (53)	0 %	0.41 %
Enrollment in other studies	2 % (43)	1 %	1.27 %
Compliance with protocol	1 % (28)	0 %	0.71 %
Lifestyle Choices			
Addictive behavior	2 % (43)	1 %	1.42 %
Bedtime	-	-	0 %
Exercise	-	-	0 %
Diet	-	-	0.14 %
Provider Characteristics			
Specialty	2 % (62)	-	-
Geographic location of practice	0.3 % (8)	-	-
Practitioner Preference	0.2 % (6)	-	-
Caregiver attribute			
Caregiver attribute	1 % (11)	-	-

^a^Percentages may sum to more than 100 % or greater that the subcategory percentage because a CES may be counted in multiple categories

## Discussion

Computer-interpretable representations of eligibility criteria have the potential to support multiple clinical research functions such as automated screening of patients for clinical trial eligibility from data already existing in the EHR and identification of patients who can benefit from the findings of existing studies. We aimed to quantify the proportion of eligibility criteria and their constituent CES that are likely to be addressable by structured data items in the EHR to understand the feasibility of automatically screening patients and identifying patients similar to a study population in the domain of primary care. In order to validate our findings, we also compared the content of eligibility criteria from clinical trials in the primary care domain to studies reported in the literature that examined the eligibility criteria used by trials conducted in tertiary care settings.

Our analysis found that 74 % of CES from these primary care studies were likely to be addressable using data elements in a typical integrated EHR. Criteria elements based on provider or investigator non-clinical judgment were considered most unlikely to be present in the EHR. Examples of this type of CES are “Considered by the GP to be unsuitable for the project” and “Not able to comply with the requirements of the protocol and therapy program, in the opinion of the assessor”. Criteria based on specific patient or caregiver preferences were also considered unlikely present in the EHR, such as: “Wishing to have support to become more active” and “Wishing to get out of the house more often.”

Eligibility criteria from 79 % of the reviewed primary care trials were judged likely to have at least 60 % of their constituent CESs addressable by EHR data elements and 14 % of trials had all of their CES satisfiable using such data. This data indicates that while EHR data may be quite useful for identifying patient cohorts for such trials, EHR data alone is often insufficient to identify an individual patient as a suitable trial subject. Most often additional screening must be done. However, that screening can be more efficient since it can be applied to a smaller subject pool that may already meet many of the criteria for inclusion in a study. The ability to evaluate more than three-quarters of CES from a sample of primary care studies using data available in an EHR confirms the feasibility of the EHR-based patient eligibility screening in similar clinical trials. In Comparison to Köpcke et al., our assessment for availability of CES as structured data elements in the EHR was higher (74 % vs. 55 %). Köpcke et al. also assessed the completeness of documentation of clinical data elements in the EHRs of actual patients and found that only 64 % of clinical data were documented. Köpcke et al. estimated that total completeness of EHR data for recruitment purposes was 35 %. Our study did not assess the completeness of documentation of the needed clinical data in the EHR needed for trial recruitment but this would be a valuable future research work.

Validation of this study’s classification of eligibility criteria was accomplished by comparison to those reported by Weng et al. and Köpcke et al. [21, 22]. Both of the comparison studies analyzed eligibility criteria from clinical trials in tertiary care settings. Weng’s categories (Table 1) accounted for the majority of the eligibility criteria elemental statement (CES) in our study with the exception of the criteria related to practice/practitioner, and caregiver attributes. Compared to Weng’s and Köpcke’s studies, this study of primary care trials found fewer occurrences of CES related to cancer (0.3 % vs. 12 % vs. 4 %), medications (10 % vs. 17 % vs. 7 %), laboratory results (5 % vs. 14 % vs. 19 %), and disease staging (1 % vs. 6 % vs. 2 %) respectively. We also found more occurrences of CES related to diseases, symptoms and signs (36 % vs. 28 % vs. 23 %) and patient non-clinical characteristics such as age (7 % vs. 2 % vs. 3 %), literacy (3 % vs. 0 % vs. 0.3 %),and patient preferences (5 % vs. 1 % vs. 0.5 %) respectively. Even though these small differences were observed, it is reasonable to conclude that Weng’s categories are a reasonable classification of primary care study CESs. It seems evident from our analysis that eligibility criteria for primary care trials are similar to the larger group of trials reported in the literature but also exhibit differences that mark them as subset of trials with some unique properties. Therefore the results reported have validity, as they are similar to what was reported for a larger, general collection of clinical trials.

It should be obvious that one limitation of this study is that it is likely that there is variation in the actual percentage of CES that are addressable among specific EHR systems depending on the actual discrete data elements used. We based our assessment on shared inpatient/ambulatory EHR systems. Our most recent experience is with the EHR system Epic (Epic, Verona, WI) operated at the University of Minnesota and the University of Michigan health systems. We believe that our conclusions are generalizable to EHRs similar to the ones we considered but may not necessarily apply to isolated EHR systems with a narrower focus. Another limitation of the study is that the assessment of whether a CES was addressable by structured data in the electronic health was subjective. However it should be noted that both individuals are experienced with EHRs, had advanced training in biomedical informatics and are either employed full-time in implementation and maintenance of an EHR system or a faculty member in health informatics who works with EHRs at several different organizations.

There also may be differences between definition of primary care in the United States and in the United Kingdom. The eligibility criteria used by our study were taken from the UKCRN Study Portfolio that defines primary care as the “care that describes range of services that are normally the first point of contact for participants”. It defines primary care studies as “studies that take place partially or wholly in primary care settings. This means it covers wide spectrum of diseases, conditions, and includes studies of disease prevention, health promotion, screening, early diagnosis, as well as management of long-conditions. It also includes studies on vaccines and palliative care” [25]. In the US, primary care physicians include family practice, geriatrics, general practice, general internal medicine, and general pediatrics [26]. The definitions used by the UKCRN study portfolio for primary care suggest that the concept of primary care in the UK is similar to the US but may have some differences. To the extent that the types of care are similar in these two settings our findings may also be, applicable to the US.

Having established that data are potentially available in an EHR is only a small step of the way to operationalizing the finding of eligible patients. The simplest means is for researchers to work with EHR system vendors or practices to create templates and search strategies specific to each EHR system. This may work well where a single vendor can cover all the trial centers, but research is increasingly large-scale and multi-national so standards for search expressions and data elements are required. The CDISC Study Data Model [13], the EU TRANSFoRm Clinical Research Information Model [11], the Electronic Health Record for Clinical Research project (EU EHR4CR) [27], and the National Patient-Centered Clinical Research Network (PCORnet) initiative [28], are all approaches to addressing the search expression problem. The profusion of models in different domains is largely led by the different data and data constraints in each domain. In particular temporal constraints on tissue diagnoses are prevalent in cancer trials and geographical and patient demographic characteristics in primary care studies. As for the data elements themselves, a simple term is often insufficient as terminologies rarely map one-to-one and differences in granularity and in the additional context of measurements such as blood pressure (clinic versus ambulatory) and lab values (pre or post therapy) mean that very careful thought needs to go into the division of concepts between CES and individual data elements [29]. TRANSFoRm uses a core Clinical Data Integration Model, expressed as ontology to deal with this issue [30], but it is clear that much detailed work is required before we can achieve a state of generalizable computable eligibility statements linked to a wide range of EHR systems.

## Conclusions

A large proportion of the data about patients that are needed to apply eligibility criteria can be found as structured data elements in an electronic health record. Use of this data can frequently expedite the screening process for enrolling subjects and in a small proportion of trials be entirely sufficient. Careful design of electronic health record systems that include data elements representing the content categories described by our study and similar studies will facilitate integration with clinical trial management systems, and improve patient care and clinical research.

## Abbreviations

CDISC: Clinical data interchange standards consortium
CES: Criteria elemental statement
CRIM: Clinical research information model
CTA: Clinical trial alerts
EHR: Electronic health record
EHR4CR: Electronic health record for clinical research
EudraCT: European clinical trials database
GP: General practitioner
NIHR: UK National institute for health research
OCRe: Ontology of clinical research
PCORnet: National patient-centered clinical research network
RCT: Randomized controlled trial
TRANSFoRm: Translational research and patient safety in Europe
UKCRN: UK Clinical research network
UMLS: Unified medical language system
